# Re-description, diet, and trophic niche overlap of three syntopic anuran species (Amphibia, Anura) in the Kim Bang Proposed Species and Habitat Conservation Area, Vietnam

**DOI:** 10.3897/zookeys.1277.185967

**Published:** 2026-04-17

**Authors:** Vien Hong Thi Nguyen, Cuong The Pham, Truong Quang Nguyen, Minh Le, Minh Duc Le, Anh Van Pham

**Affiliations:** 1 Faculty of Resources and Environment, University of Sciences, Thai Nguyen University, Phan Dinh Phung Ward, Thai Nguyen, Vietnam Faculty of Resources and Environment, University of Sciences, Thai Nguyen University Thai Nguyen Vietnam; 2 Institute of Biology, Vietnam Academy of Science and Technology, 18 Hoang Quoc Viet Road, Hanoi 10072, Vietnam Central Institute for Natural Resources and Environmental Studies, Vietnam National University Hanoi Vietnam; 3 Graduate University of Science and Technology, Vietnam Academy of Science and Technology, 18 Hoang Quoc Viet Road, Hanoi 10072, Vietnam Faculty of Environmental Sciences, University of Science, Vietnam National University Hanoi Vietnam; 4 Central Institute for Natural Resources and Environmental Studies, Vietnam National University, Hanoi, 19 Le Thanh Tong, Hanoi, Vietnam Graduate University of Science and Technology, Vietnam Academy of Science and Technology Hanoi Vietnam; 5 Department of Herpetology, American Museum of Natural History, Central Park West at 79th Street, New York 10024, USA Institute of Biology, Vietnam Academy of Science and Technology Hanoi Vietnam; 6 Faculty of Environmental Sciences, University of Science, Vietnam National University, Hanoi, 334 Nguyen Trai Road, Hanoi 11416, Vietnam Department of Herpetology, American Museum of Natural History New York United States of America

**Keywords:** Coleoptera, distribution, Hymenoptera, Isoptera, Ninh Binh Province, prey items, stomach contents

## Abstract

As a result of our field surveys in April and July 2025 in the Kim Bang Proposed Species and Habitat Conservation Area, we provide an extended morphological description of three syntopic anuran species, namely *Microhyla heymonsi*, *M. mukhlesuri*, and *Occidozyga lingnanica* based on newly collected specimens. In addition, we present data on the diet and trophic niche overlap of these syntopic anuran species. Using the stomach-flushing method, we analyzed stomach contents of 26 individuals of *M. heymonsi*, 42 individuals of *M. mukhlesuri*, and 48 individuals of *O. lingnanica*. We found 878 prey items, belonging to five insect orders (Coleoptera, Hemiptera, Hymenoptera, Isoptera, Thysanoptera) and Araneae in the diet of *Microhyla mukhlesuri*; 501 prey items, belonging to three insect orders (Coleoptera, Hymenoptera, Isoptera) in *M. heymonsi*; and 280 prey items, belonging to eight insect orders (Coleoptera, Diptera, Hemiptera, Hymenoptera, Isoptera, Lepidoptera, Orthoptera, Thysanoptera), Decapoda, Isopoda, Gastropoda, Araneae, and Gobiiformes in *Occidozyga lingnanica*. The highest frequency of occurrence (%F) of prey items identified in *O. lingnanica* was Coleoptera (48.25%), whereas the highest frequency of occurrence of prey items identified in *M. heymonsi* and *M. mukhlesuri* was Isoptera (55.81% and 40.91%, respectively). The trophic niche breadth was highest in *O. lingnanica* (*B_sta_* = 0.33), followed by *M. mukhlesuri* (*B_sta_* = 0.06), and the lowest values of niche breadth was recorded for *M. heymonsi* (*B_sta_* = 0.02). The overlap of trophic niche (*O_jk_*) was 97% between *M. mukhlesuri* and *M. heymonsi*, 49% between *M. heymonsi* and *O. lingnanica*, and 47% between *O. lingnanica* and *M. mukhlesuri*.

## Introduction

Basic data on the ecology of organisms facilitate the construction of hypotheses related to niche partitioning and general ecology (Farina et al. 2023). To reduce competition, co-existing species may use resources differently to increase their chances of survival (Pianka 1974). Food is an important niche axis for partitioning among coexisting anurans (Toft 1980), and food niche overlap has been used to hypothesize potential interactions between sympatric species (Hirai and Matsui 2000). The number of studies on the diet of anurans in Vietnam has increased in recent years (Pham et al. 2019b, 2019a, 2023, 2024b, 2024a). However, only a few studies focus on a comparative approach to the species’ diet, viz. Pham et al. (2022) analyzed food types of *Microhyla butleri* Boulenger, 1900 and *M. heymonsi* Vogt, 1911 from Son La Province, and found up to 97% similarity between two species with regard to the trophic niche overlap. Trinh et al. (2023) examined segregation in diet composition of two syntopic tree frog species, *Hyla simplex* Boettger, 1901 and *Polypedates megacephalus* Hallowell, 1861, in Ben En National Park (NP), Thanh Hoa Province and found similarity between two species with ~ 40% trophic overlap.

In this study, we test the hypothesis by analyzing diet compositions of three sympatric frogs, *Microhyla heymonsi* Vogt, 1911, *M. mukhlesuri* Hasan, Islam, Kuramoto, Kurabayashi & Sumida, 2014, and *Occidozyga lingnanica* Lyu & Wang, 2022, that occur in syntropy in two streams from Kim Bang Proposed Species and Habitat Conservation Area (SHCA), Ninh Binh Province, northern Vietnam. The Lingnan Floating Frog *Occidozyga lingnanica* is known from China (Guangdong and Hainan provinces) and Vietnam (Ninh Binh and Thanh Hoa provinces) (Frost 2025). The Heymon’s Rice Frog *Microhyla heymonsi* occurs in Taiwan, southern China (from Zhejiang to Yunnan, including Hainan), northern Vietnam (Lao Cai and Tuyen Quang provinces in the north, south to Kien Giang and Ca Mau provinces), Myanmar (Bago, Kachin, Kayah, Shan, Yangon, Mon, Tanintharyi), through to Thailand, West Malaysia (Pham et al. 2022; Frost 2025). The Ornamented Pygmy Frog *Microhyla mukhlesuri* is known from southwestern of the Red River of Yunnan (China) and Vietnam, Laos, Myanmar, India, eastern Bangladesh, Thailand, Malaysia, and Singapore. This study aims to investigate the diet composition and trophic niche overlap of three syntopic anuran species in the Kim Bang SHCA, northern Vietnam.

## Material and methods

### Sampling

Field surveys were conducted in Kim Bang SHCA, Ninh Binh Province, Vietnam from 21 to 28 April and 22 to 30 July 2025 by Nguyen QT, Pham TC, Phan QT, and Pham VA. The coordinates (WGS 84) and elevations were determined by using the GPS Garmin 62SX. Frogs were collected by hand between 20:30 and 23:00 h following the guidelines approved by the American Society of Ichthyologists and Herpetologists for animal care (Beaupre et al. 2004). In this study, we used the stomach-flushing technique to obtain stomach contents without sacrificing them (Griffiths 1986; Leclerc and Courtois 1993; Solé et al. 2005). Each specimen was stomach-flushed only once following the guidelines approved by the American Society of Ichthyologists and Herpetologists for animal care (Beaupre et al. 2004). The water for flushing was taken from the streams where the frogs were captured and used after filtration. After stomach-flushing, frogs were monitored for vigor and body conditions and released within 30 min at the place of capture. Prey items were preserved in 70% ethanol. Frogs were subsequently released at the collecting site after measurements of snout-vent length (SVL), mouth width (MW) with digital calipers to the nearest 0.01 mm taken and body mass (BM) with an electronic balance.

For taxonomic identification, two individuals of each species were collected for voucher specimens. After having been photographed in life, animals were anesthetized and euthanized in a closed vessel with a piece of cotton wool containing ethyl acetate, fixed in 85% ethanol and subsequently stored in 70% ethanol (Simmons 2002). Specimens were deposited in the collections of the Faculty of Environmental Sciences, University of Science, Vietnam National University, Hanoi (VNU-HUS).

### Molecular data

For voucher specimens, one individual of each species was collected for DNA analyses for species identification. We used the protocols of Le et al. (2006) for DNA extraction, amplification, and sequencing. A fragment of the mitochondrial gene, 16S ribosomal RNA, of ~ 550–560 bp was amplified and sequenced using the primer pair LR N 13398 (5'-CGCCTGTTTACCAAAAACAT-3'; forward), LR J 12887 (5'-CCGGTCTGAACTCAGATCACGT-3'; reverse) (Simon et al. 1994). Sequences were compared with those from GenBank using Basic Local Alignment Search Tool (BLAST) searches.

### Stomach content analysis

Prey items were identified under a microscope (Olympus SZ 700) based on identification keys (i.e. Naumann et al. 1991; Thai 2003; Johnson and Triplehorn 2005; Brusca et al. 2016). The maximum length (*L*) and width (*W*) of each prey item were measured to the nearest 0.1 mm using either calipers or a calibrated ocular micrometer fitted to a microscope. The volume (*V*) of prey items was calculated using the formula for a prolate spheroid (*V* = 4π / 3 × (*L* / 2) × (*W* / 2)^2^ (mm^3^), *π* = 3.14 (Pham et al. 2024a). The index of relative importance (*IRI*) was used to determine the importance of each food category. This index provides a more informed estimation of prey item consumption than any of the three components alone by using the following formula: *IRI* = (%*F* + %*N* + %*V*) / 3, where *F* is the frequency of prey occurrence in stomachs and *N* is the total number of prey items concerning all prey items (Pham et al. 2024b). We used the reciprocal Simpson’s heterogeneity index, *1-D*, to calculate dietary heterogeneity: *D* = ∑[*n*_i_(*n*_i_ – 1)] / [*N*(*N* – 1)]. Where ni is the number of prey items in the i^th^ taxon category and *N* is the total number of prey items (Krebs 1999).

To estimate prey evenness, we used Shannon’s index of evenness. Evenness is calculated from the equation: *J*' = *H*' / Hmax = *H*' / ln *S* (Pham et al. 2024b). The maximum diversity (Hmax) that could occur is that which would be found in a situation in which all taxa had equal abundance (H' = Hmax = ln S), S is the total number of prey taxa, and *H*' is the Shannon–Weiner index of taxon diversity. The value of *H*' is calculated from the equation: *H*' = –∑(*P*_i_ × ln *P*_i_), where the quantity *P*_i_ is the proportion of total prey items belonging to the i^th^ taxon for the total prey items of the sample (Pham et al. 2019b).

To test for differences in the diet composition between species, we performed an analysis of similarity (ANOSIM) on volume and abundance matrices of prey items (Oksanen et al. 2026). ANOSIM provides an r-index with values between –1 and 1, *r* values close to 1 suggest dissimilarity between groups, and values close to –1 indicate similarity. Significance was assessed with a *p* < 0.05 (Chapman and Underwood 1999). We calculated the dissimilarity matrix with the Bray-Curtis distance (Sánchez-Loja et al. 2024). We then used a non-metric multidimensional scaling (NMDS) to visualize prey assemblages of frog species in a two-dimension plot (Saporito et al. 2012; Moskowitz et al. 2018; Sánchez-Loja et al. 2024). An NMDS provides a Stress value that ranges from 0 to 1. Stress values < 0.2 suggest that distances in the plot are good representations of distances in the assemblage matrix (Sánchez-Loja et al. 2024). All statistical analyses were conducted in R v4.5.2 with the vegan package (Oksanen et al. 2026; R Core Team 2025).

The Levin’s Standardized Niche Breadth Index (*B_sta_*) ranges from 0 to 1 and is calculated according to the following equation (Krebs 1999; Farina et al. 2023): *B_sta_* = (B-1)/(n-1), where n is the number of resources registered in the diet (prey categories), and B = 1 / Σ*pi*^2^, *p* represents the proportion of individuals of a given prey category (i) found in the diet. Values near 0 indicate a specialist diet (narrow niche breadth), while values near 1 indicate a generalist diet (wide niche breadth). *B_sta_* was used to facilitate comparisons of trophic ecology between species.

For the analysis of overlap in niche dimensions and/or the degree of similarity between diets of the species pair, we used Pianka’s niche overlap index (*O*_jk_) (Pianka 1973), with values ranging from 0 (no overlap) to 1 (complete overlap) (Krebs 1999). This overlap was calculated using the following expression:

$$O_{jk}=\frac{\sum_{i=1}^{n}P_{\mathrm{ij}}P_{ik}}{\sqrt{\sum_{i=1}^{n}P_{\mathrm{ij}}^{2}\sum_{n=1}^{n}P_{ik}^{2}}}$$

Where *O*_jk_ represents the index of overlap of Pianka’s niche between species j and k; *P_ij_* is the proportion of prey i^th^ in the total prey categories used by species j; *P_ik_* is the proportion of prey i^th^ in total prey categories used by species k; and *n* is the total number of prey categories for species j and k.

We performed analysis of Kendall’s tau-b and multiple regression using SPSS 20.0 (SPSS Inc., Chicago, Illinois, USA), with the significance level set to *p* < 0.05 for all analyses. Data are presented as mean ± standard deviation (SD) unless otherwise noted. Kendall’s tau-b statistics were used to examine relationships between the frog SVL and the prey volume of each individual (Pham et al. 2024b). Multiple regression analysis was used to examine the relationships between body size (SVL, MW, and BM), prey width, and prey volume (Pham et al. 2025).

## Results

### Taxonomic identification

*Microhyla heymonsi* Vogt, 1911

**Figure 1.**
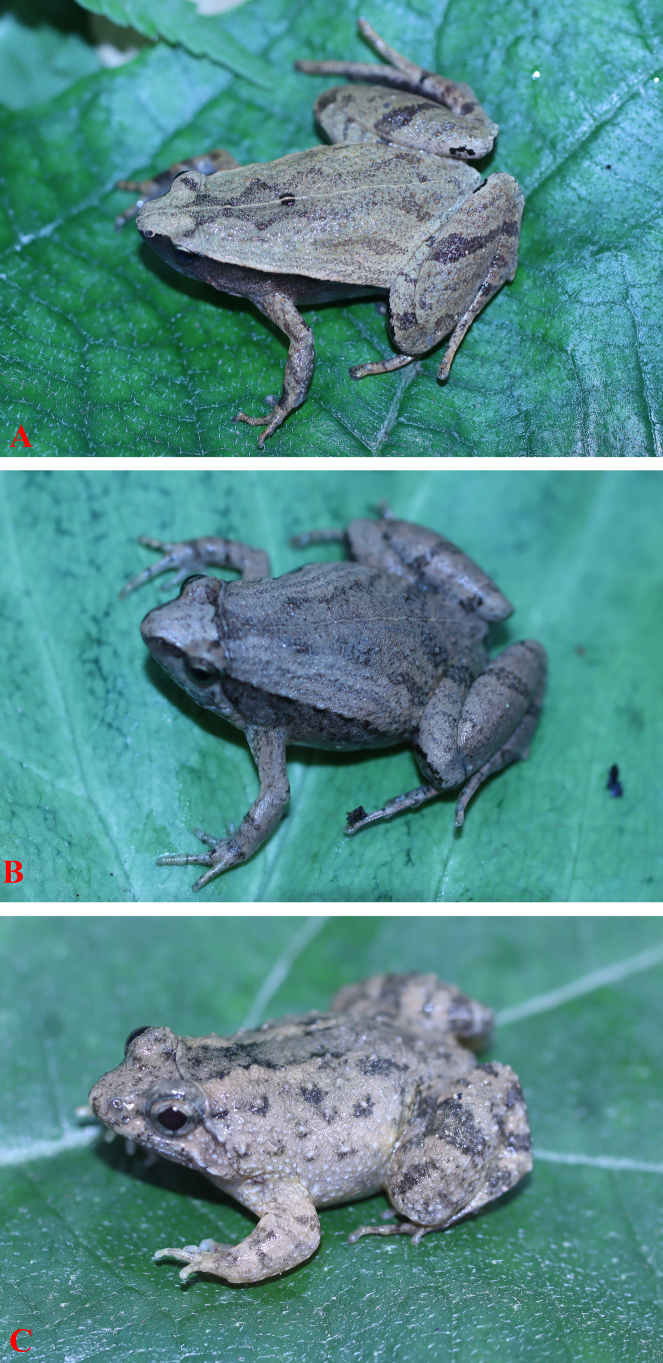
Adult male of frogs. **A**. *Microhyla heymonsi*; **B**. *M. mukhlesuri*, and **C**. *Occidozyga lingnanica*.

**Figure 2.**
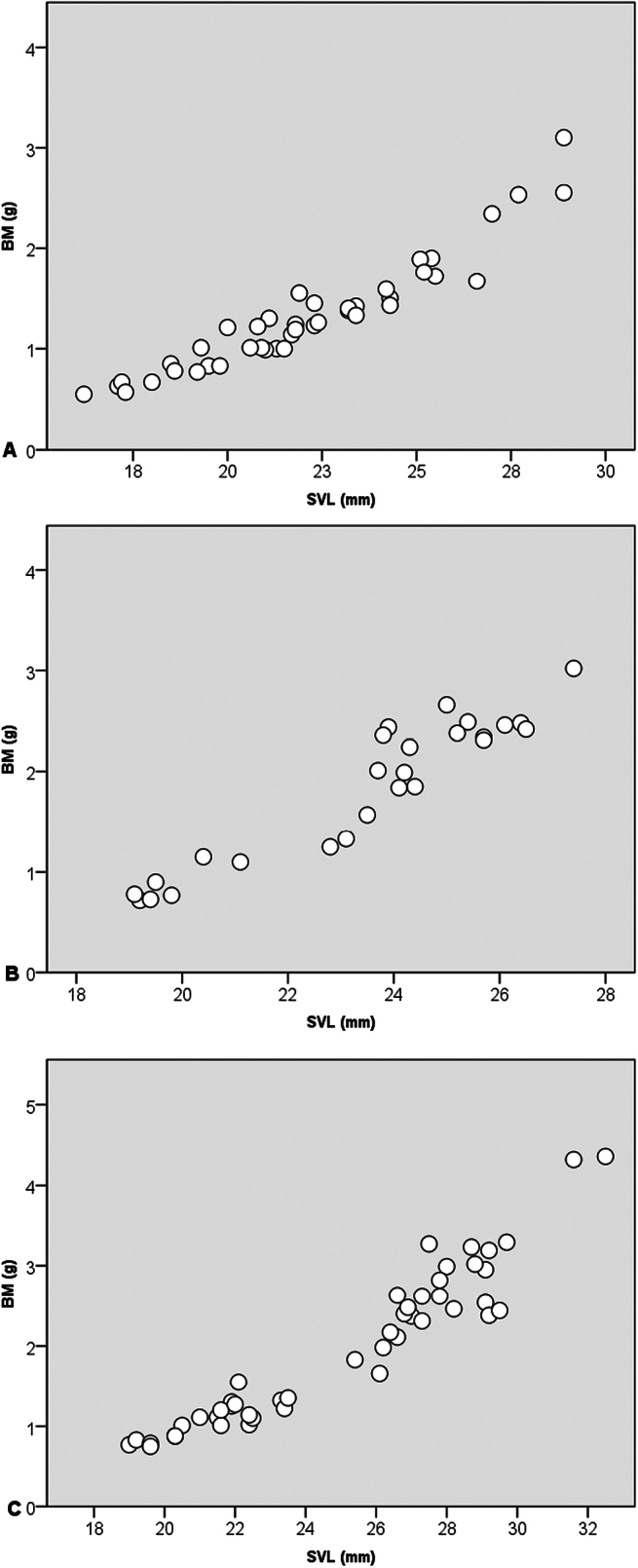
Dispersion diagrams from Pearson’s correlations between snout vent length and body mass. **A**. *Microhyla mukhlesuri*; **B**. *M. heymonsi*, and **C**. *Occidozyga lingnanica*.

Morphological characters of the frogs from Kim Bang SHCA agreed well with the descriptions of Bourret (1942), Manthey and Grossmann (1997), and Hecht et al. (2013): SVL min–max: 19.1–21.1 mm, mean ± SD: 19.85 ± 0.77 mm, HL 5.3–6.7 mm (6.02 ± 0.56 mm), MW 4.9–5.8 mm (5.27 ± 0.35 mm), BM 0.72–1.15 g (0.90 ± 0.20 g), *n* = 6, in males; SVL min–max: 19.4–27.4 mm, mean ± SD: 24.53 ± 1.73 mm, HL 5.9–8.1 mm (7.25 ± 0.53 mm), MW 5.1–6.7 mm (6.21 ± 0.36 mm), BM 0.73–3.02 g (2.11 ± 0.55 g), *n* = 20, in females. There were strong positive correlations between the morphological measurements (SVL and MW: *r* = 0. 839, *p* < 0.001; SVL and BM: *r* = 0.945; *p* < 0.001; MW and BM: *r* = 0. 827, *p* < 0.001) (Table 1, Figs 1A, 2A).

**Table 1.** Measurements (in mm) and weight (in g) of three frog species in the Kim Bang SHCA, Ninh Binh Province, Vietnam.

	*Microhyla mukhlesuri* (*n* = 42)	*M. heymonsi* (*n* = 26)	*Occidozyga lingnanica* (*n* = 48)
SVL	22.1 ± 3.2	23.45 ± 2.54	24.91 ± 3.7
	16.2–28.9	19.1–27.4	19–32.5
HL	6.8 ± 0.86	6.96 ± 0.74	8.7 ± 1.05
	5–9	5.3–8.1	6.4–10.6
MW	5.64 ± 0.74	5.99 ± 0.54	7.61 ± 1.0
	4–7.5	4.9–6.7	5.8–9.5
BM	1.32 ± 0.56	1.87 ± 0.7	1.96 ± 0.96
	0.55–3.1	0.72–3.02	0.75–4.36

One voucher specimen (HUS 2026 A.02, adult male): snout obtusely pointed, somewhat longer than eye; interorbital distance broader than upper eyelid; tympanum invisible; vomerine teeth absent; tongue roundly spatulate and free at the rear half of its length; external vocal sac present in male. Forelimb slender; fingers free of webbing and tips not swollen. Hindlimb slender, tibia longer than thigh; toe tips round, not swollen, webbed at base; subarticular tubercle present; inner and outer metatarsal tubercles present; tibio-tarsal articulation reaching tip of snout. Dorsal skin smooth, dorsolateral edges not sharp; ventral skin smooth. Dorsal surface of head and body pale grey, with a white stripe from tip of snout to cloaca, and a small dark spot in the center of the back; lateral side of head and flank dark brown to black; limbs with thin transverse bars; ventral surface white to grey (Fig. 1A).

A 549-bp long sequence (GenBank accession number PZ245531) obtained from a specimen (HUS 2026 A.02) from Kim Bang SHCA was 98.91% similar to that with GenBank accession number OM387068 of *Microhyla heymonsi* collected in northern Vietnam.

#### *Microhyla mukhlesuri* Hasan, Islam, Kuramoto, Kurabayashi & Sumida, 2014

Morphological characters of the frogs from Kim Bang SHCA agreed well with the description of Hasan et al. (2014): SVL min–max: 16.2–25.2 mm, mean ± SD: 20.69 ± 2.22 mm, HL 5.0–7.3 mm (6.48 ± 0.64 mm), MW 4.0–6.3 mm (5.35 ± 0.57 mm), BM 0.55–1.76 g (1.08 ± 0.32 g), *n* = 31, in males; SVL min–max: 23.2–28.9 mm, mean ± SD: 26.08 ± 1.90 mm, HW 6.3–8.1 mm (6.99 ± 0.52 mm), MW 5.6–7.5 mm (6.43 ± 0.56 mm), BM 1.40–3.10 g (2.00 ± 0.55 g), *n* = 11, in females. There were strong positive correlations between the morphological measurements (SVL and MW: *r* = 0. 905, *p* < 0.001; SVL and BM: *r* = 0. 943; *p* < 0.001; MW and BM: *r* = 0.897, *p* < 0.001) (Table 1, Figs 1B, 2B).

One voucher specimen (HUS 2026 A. 01, adult male): snout round, longer than eye; vomerine teeth absent; tongue roundly spatulate and free at the rear half of its length; external vocal sac present in male. Forelimb slender; fingers free of webbing and tips not swollen. Hindlimb slender, tibia longer than thigh; toe tips round, not swollen, webbed at base; subarticular tubercle present; inner and outer metatarsal tubercles present; tibio-tarsal articulation reaching behind the eye. Dorsal skin smooth, dorsolateral ridges discontinuous; ventral skin smooth. Dorsal surface of head and body grey with a dark X-shaped mark on the dorsum, arising from the eyes to the groin; limbs with dark transverse bars; ventral surface whitish, throat and chest mottled with dark brown (Fig. 1B).

A 556-bp long sequence (GenBank accession number PZ245533) obtained from a specimen (HUS.2026 A.01) from Kim Bang SHCA was 97.66% similar to that with GenBank accession number AB543609 of *Microhyla mukhlesuri* collected in Raozan, Chittagong Distrct, Bangladesh (Hasan et al. 2014).

#### *Occidozyga lingnanica* Lyu & Wang, 2022

Morphological characters of the frogs from Kim Bang SHCA agreed well with the description of Lyu et al. (2022): SVL min–max: 19.0–27.8 mm (mean ± SD: 23.65 ± 2.77 mm), HL 6.4–10.0 mm (8.43 ± 0.84 mm), MW 5.8–9.0 mm (7.37 ± 0.83 mm), BM 0.75–2.62 g (1.55 ± 0.60 g), *n* = 33, in males; SVL min–max: 26.6–32.5 mm (mean ± SD: 28.97 ± 1.54 mm), HL 8.9–10.6 mm (9.72 ± 0.45 mm), MW 8.1–9.5 mm (8.64 ± 0.40 mm), BM 2.38–4.36 g (3.07 ± 0.60 g), in females, *n* = 15. There were strong positive correlations between the morphological measurements (SVL and MW: *r* = 0.924, *p* < 0.001; SVL and BM: *r* = 0.950; *p* < 0.001; MW and BM: *r* = 0.886, *p* < 0.001) (Table 1, Figs 1C, 2C).

One voucher specimen (adult male): head longer than wide; snout round in dorsal view and profile, longer than eye; interorbital distance broader than upper eyelid; tympanic rim indistinct; vomerine teeth absent; tongue round posteriorly; external vocal sac present in male. Forelimb slender; fingers free of webbing, tips round and not dilated; relative finger lengths II=I<IV<III; nuptial pad present in male. Hindlimb slender, tibia longer than thigh; relative lengths I<II<V<III<IV; toe tips round, dilated into round disks; toes fully webbed; subarticular tubercles present; inner and outer metatarsal tubercles present; tibio-tarsal articulation reaching at the posterior margin of supratympanic fold. Dorsal surface of body and limb rough, with large tubercles, flanks with tubercles; a faint fold across head between orbits; supratympanic fold distinct; ventral surface with flat tubercles; a fold across breast; and dense granules on the ventral tarsi. Dorsal surface greyish brown with irregular black speckles; a narrow transverse dark bar between orbits; mid-dorsal stripe yellowish brown but indistinct; limbs with dark brown transverse bars; throat dark grey with white mottling; chest and belly uniform creamy white (Fig. 1C).

A 557-bp long sequence (GenBank accession number PZ245534) obtained from a specimen (HUS 2026 A.03) from Kim Bang SHCA was 98.91% similar to that with GenBank accession number ON615087 of *Occidozyga lingnanica* collected in Zhuhai, Guangdong, China.

#### Diet

We analyzed the stomach contents of 116 individuals. Of these, 26 were *Microhyla heymonsi* (501 items), 42 *M. mukhlesuri* (containing 878 food items), and 48 *Occidozyga lingnanica* (280 items). The Simpson’s heterogeneity index in *O. lingnanica* (*1* – *D* = 0.91 with an evenness index of *J’* = 0.82) was higher than that in *M. heymonsi* (*1* – *D* = 0.39 with an evenness index of *J’* = 0.27) and *M. mukhlesuri* (*1* – *D* = 0.15 with an evenness index of *J’* = 0.12) (Table 2). The average number of prey items per stomach in *M. mukhlesuri* was 20.9 ± 22.58 items (ranging from 3–125), in *M. heymonsi* was 19.27 ± 15.57 items (ranging from 1–60), and *O. lingnanica* was 5.83 ± 4.42 items (ranging from 1–19 (Table 3).

**Table 2.** Simpson’s Index of Diversity, Shannon’s Evenness, and Levin’s Standardized Niche Breadth Index among species in the diet of frogs from Kim Bang SHCA.

Species	Simpson’s heterogeneity index (1-*D*)	Shannon’s evenness (*J*’)	Levin’s Standardized Niche Breadth Index (*B_sta_*)
*Microhyla mukhlesuri*	0.39	0.27	0.06
*Microhyla heymonsi*	0.15	0.12	0.02
*Occidozyga lingnanica*	0.91	0.82	0.33

**Table 3.** Summary (Mean, and range) of the prey item number (*N*), width (*W*, in mm), length (*L*, in mm), and volume (*V*, in mm^3^) data for frogs.

	*Microhyla heymonsi* (*n* = 26)	*M. mukhlesuri* (*n* = 42)	*Occidozyga lingnanica* (*n* = 48)
N	19.27 ± 15.57	20.9 ± 22.58	5.83 ± 4.42
	1–60	3–125	1–19
	(*n* = 501)	(*n* = 878)	(*n* = 280)
W	1.09 ± 0.19	0.96 ± 0.21	1.35 ± 0.72
	0.5–2.0	0.5–3.0	0.5–5.0
L	2.51 ± 1.28	2.03 ± 1.17	3.66 ± 3.14
	0.5–5.0	0.5–12.0	0.5–20.0
V	32.42 ± 34.57	22.86 ± 32.52	40.8 ± 50.53
	0.92–121.41	1.11–143.66	1.05–266.59

We identified eight insect orders (Coleoptera, Diptera, Hemiptera, Hymenoptera, Isoptera, Lepidoptera, Orthoptera, Thysanoptera), Decapoda, Isopoda, Gastropoda, Araneae, Gobiiformes in the diet of *Occidozyga lingnanica*; five insect orders (Coleoptera, Hemiptera, Hymenoptera, Isoptera, Thysanoptera) and Araneae in *Microhyla mukhlesuri*; and three insect orders (Coleoptera, Hymenoptera, Isoptera) in *M. heymonsi*. The highest frequency of occurrence (%F) of prey items identified in *O. lingnanica* was Coleoptera (48.25%), followed by Orthoptera (9.65%), Hymenoptera and Isoptera (7.89%), and Hemiptera (6.14%), whereas the highest frequency of occurrence (%F) of prey items identified in *M. heymonsi* and *M. mukhlesuri* is Isoptera (55.81% and 40.91%, respectively), followed by Hymenoptera (25.58% and 31.82%, respectively), and Coleoptera (18.6% and 23.86%, respectively). In the comparisons by the IRI (%) in *O. lingnanica* was Coleoptera (47.2%), followed by Orthoptera (12.16%), and Hymenoptera (8.47%), in *M. mukhlesuri* was Isoptera (65.13%), followed by Hymenoptera (20.65) and Coleoptera (12.9%), and in *M. heymonsi* was Isoptera (80.99%) (Table 4).

**Table 4.** Dietary composition (%) of frogs: frequency of occurrence (*F*), numeric proportion (*N*), volume proportion (*V*), and overall importance (*IRI*) value of each prey taxon.

	*Microhyla mukhlesuri* (*n* = 42)				*M. heymonsi* (*n* = 26)				*Occidozyga lingnanica* (*n* = 48)			
Prey category	%F	%N	%V	IRI	%F	%N	%V	IRI	%F	%N	%V	IRI
** Decapoda **	–	–	–	–	–	–	–	–	**1.75**	**1.07**	**6.31**	**3.04**
Atyidae	–	–	–	–	–	–	–	–	1.75	1.07	6.31	3.04
** Isopoda **	–	–	–	–	–	–	–	–	**1.75**	**1.07**	**5.26**	**2.70**
Armadillidiidae	–	–	–	–	–	–	–	–	1.75	1.07	5.26	2.70
** Gastropoda **	–	–	–	–	–	–	–	–	**6.14**	**3.21**	**2.44**	**3.93**
** Araneae **	**1.14**	**0.11**	**0.05**	**0.43**	**–**	**–**	**–**	**–**	**3.51**	**1.43**	**1.34**	**2.09**
** Coleoptera **	**23.86**	**3.99**	**10.86**	**12.90**	**18.60**	**1.60**	**1.52**	**7.23**	**48.25**	**43.57**	**49.79**	**47.20**
Carabidae	–	–	–	–	2.33	0.20	0.12	0.88	0.88	0.36	1.80	1.01
Curculionidae	–	–	–	–	2.33	0.20	0.09	0.87	–	–	–	–
Chrysomelidae	1.14	0.11	0.05	0.43	–	–			–	–	–	–
Dytiscidae	–	–	–	–	–	–			0.88	1.07	0.19	0.71
Hydrophilidae	6.82	1.48	0.73	3.01	2.33	0.20	0.09	0.87	6.14	3.57	2.42	4.04
Larvae Coleoptera	7.95	1.37	8.89	6.07	6.98	0.60	0.20	2.59	12.28	15.71	34.04	20.68
Nitidulidae	1.14	0.11	0.02	0.42	2.33	0.20	0.03	0.85	5.26	3.93	2.31	3.84
Phalacridae	5.68	0.80	1.10	2.52	2.33	0.20	0.99	1.17	3.51	1.43	0.27	1.73
Staphylinidae	–	–	–	–	–	–	–	–	4.39	2.86	0.69	2.64
Tenebrionidae	–	–	–	–	–	–	–	–	0.88	0.71	0.37	0.66
Other Coleoptera	1.14	0.11	0.07	0.44	–	–	–	–	14.04	13.93	7.69	11.89
** Diptera **	**–**	**–**	**–**	**–**	**–**	**–**	**–**	**–**	**4.39**	**3.57**	**4.32**	**4.09**
Larvae Diptera	–	–	–	–	–	–	–	–	1.75	2.14	4.18	2.69
Drosophilidae	–	–	–	–	–	–	–	–	1.75	1.07	0.13	0.99
Culicoidea	–	–	–	–	–	–	–	–	0.88	0.36	0.01	0.41
** Hemiptera **	**1.14**	**0.11**	**0.11**	**0.45**	–	–	–	–	**6.14**	**4.29**	**1.98**	**4.13**
Cicadellidae	–	–	–	–	–	–	–	–	0.88	0.36	0.64	0.63
Gerridae	–	–	–	–	–	–	–	–	0.88	1.43	0.29	0.87
Pentatomidae	–	–	–	–	–	–	–	–	1.75	1.07	0.86	1.23
Mesoveliidae	1.14	0.11	0.11	0.45	–	–	–	–	2.63	1.43	0.19	1.42
** Hymenoptera **	**31.82**	**19.82**	**10.32**	**20.65**	**25.58**	**6.19**	**3.52**	**11.76**	**7.89**	**6.79**	**10.72**	**8.47**
Apidae	–	–	–	–	–	–	–	–	0.88	0.36	8.02	3.08
Formicidae	31.82	19.82	10.32	20.65	25.58	6.19	3.52	11.76	7.02	6.43	2.70	5.38
** Isoptera **	**40.91**	**75.85**	**78.63**	**65.13**	**55.81**	**92.22**	**94.94**	**80.99**	**7.89**	**14.64**	**1.76**	**8.10**
Termitidae	40.91	75.85	78.63	65.13	55.81	92.22	94.94	80.99	7.89	14.64	1.76	8.10
** Lepidoptera **	**–**	**–**	**–**	**–**	**–**	**–**	**–**	**–**	**0.88**	**0.36**	**0.86**	**0.70**
Larvae Lepidoptera	–	–	–	–	–	–	–	–	0.88	0.36	0.86	0.70
** Orthoptera **	–	–	–	–	–	–	–	–	**9.65**	**18.57**	**8.26**	**12.16**
Acrididae	–	–	–	–	–	–	–	–	6.14	13.57	5.74	8.48
Gryllidae	–	–	–	–	–	–	–	–	3.51	5.00	2.52	3.68
** Thysanoptera **	**1.14**	**0.11**	**0.03**	**0.43**	**–**	**–**	**–**	**–**	**0.88**	**0.36**	**0.13**	**0.46**
Thripidae	1.14	0.11	0.03	0.43	–	–	–	–	0.88	0.36	0.13	0.46
** Gobiiformes **	**–**	**–**	**–**	**–**	**–**	**–**	**–**	**–**	**0.88**	**1.07**	**6.84**	**2.93**
Gobiidae	–	–	–	–	–	–	–	–	0.88	1.07	6.84	2.93

The trophic niche breadth was higher for *O. lingnanica* (*B_sta_* = 0.33), followed by *M. mukhlesuri* (*B_sta_* = 0.06), and the lowest values of niche breadth were recorded for *M. heymonsi* (*B_sta_* = 0.02) (Table 2).

**Figure 3.**
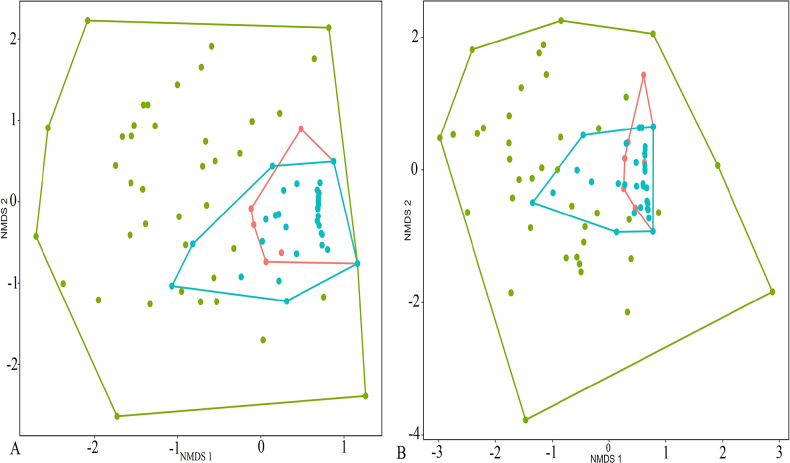
Non-metric multidimensional scaling (NMDS) analysis based on Bray-Curtis dissimilarity shows the difference of **A**. The abundance of prey consumed by *Microhyla mukhlesuri*, *M. heymonsi*, and *Occidozyga lingnanica* (Stress: 0.198), and **B**. Volume of prey consumption between *M. mukhlesuri*, *M. heymonsi*, and *O. lingnanica* (Stress: 0.182). Each point represents the diet of each frog studied (bright turquoise for *M. mukhlesuri*; pink for *M. heymonsi*, and aero blue for *O. lingnanica*.

The NMDS showed a high overlap in the diets between *M. heymonsi* and *M. mukhlesuri*; correspondingly, ANOSIM revealed no significant differences in the diet composition of the frog species for abundance (*r* = – 0.0248, *p* = 0.738), and volume (*r* = 0.0241, *p* = 0.233) data (Fig. 3A). While, the NMDS showed a slight overlap in the diets between *M. heymonsi* and *O. lingnanica* as well as *O. lingnanica* and *M. mukhlesuri*, however, ANOSIM corroborated significant differences in the diet composition of the frog species for abundance (*r* = 0.2409, *p* = 0.001; *r* = 0.3384, *p* = 0.001, respectively), and volume (*r* = 0.2426, p = 0.001; *r* = 0.3306, *p* = 0.001, respectively) data (Fig. 3B).

**Figure 4.**
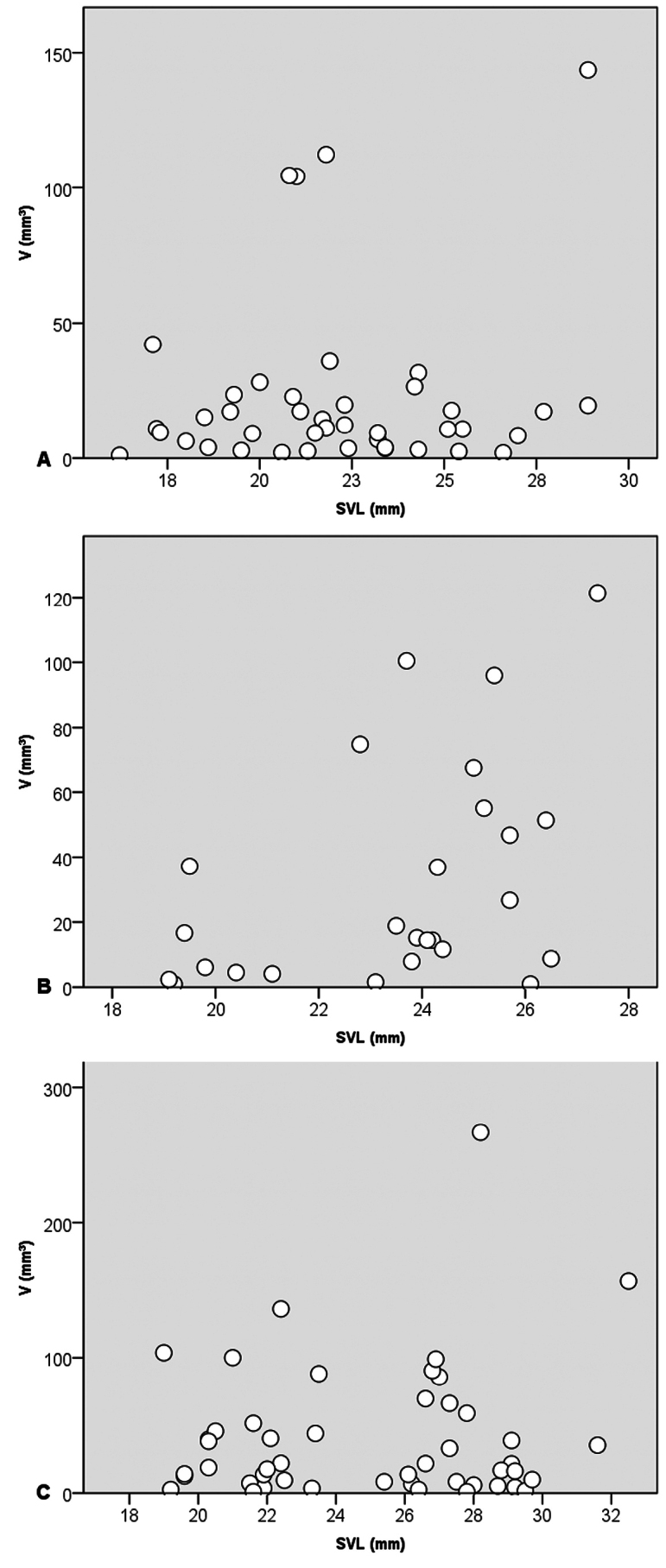
Relationships between the SVL and stomach contents volume (V). **A**. *Microhyla mukhlesuri*; **B**. *M. heymonsi*, and **C**. *Occidozyga lingnanica*.

The overlap of trophic niche (*O_jk_*) was 97% between *M. mukhlesuri* and *M. heymonsi*, 49% between *M. heymonsi* and *O. lingnanica*, and 47% between *O. lingnanica* and *M. mukhlesuri*.

The stomach contents volume (V) of *O. lingnanica* was V = 40.8 ± 50.53 mm^3^ (1.05–266.59 mm^3^, *n* = 47), in *M. mukhlesuri*, V = 22.86 ± 32.52 mm^3^ (1.11–143.66 mm^3^, *n* = 42), and in *M. heymonsi* V = 32.42 ± 34.57 mm^3^ (0.92–121.41 mm^3^, *n* = 26).

There was no correlation between SVL of *M. heymonsi*, *M. mukhlesuri*, *O. lingnanica* and the stomach contents volume (Kendall’s tau-b: *tau* = 0.299, *P* = 0.320; *tau* = 0.001, *P* = 0.991; *tau* = – 0.15, *P* = 0.88, respectively).

## Discussion

Using the stomach-flushing method, we are able to collect and determine the dietary composition of three frog species in Kim Bang SHCA, Ninh Binh Province, Vietnam. Our preliminary results showed that the diets of *M. heymonsi*, *M. mukhlesuri*, and *O. lingnanica* mainly consisted of invertebrates. A higher degree of dietary similarity was recorded between *M. heymonsi* and *M. mukhlesuri*, primarily driven by the dominance of Formicidae and Termitidae in their diets. This shared reliance on key prey groups likely underlies the substantial trophic niche overlap between these species. These results are similar to previous findings in Son La Province, Vietnam, west Java, southwestern Taiwan, northern Peninsular Malaysia of Erftemeijer (Norval et al. 2014; Hui 2015; Pham et al. 2022). Formicidae has been reported as an important prey category in the diet of many anurans (Duellman and Trueb 1994; Wells 2007; Hui 2015; Pham et al. 2022). In addition, both *M. heymonsi* and *M. mukhlesuri* occupy in similar microhabitats and ecological niches such as grasslands, shrubs in secondary forest habitats and fields, and small ponds. Such similarities in where they occur may account for the high degree of overlap in the trophic niche of the two species. However, four prey categories of Chrysomelidae, Mesoveliidae, and Thripidae were found exclusively in the diet of *M. mukhlesuri*, whereas two prey categories of Carabidae and Curculionidae were found only in the diet of *M. heymonsi*. The prey categories of rice frogs in Son La Province were similar to those in Ninh Binh Province (13 vs 12 in the latter), with Hymenoptera, Isoptera, and Coleoptera being the most important prey items (Pham et al. 2022). Diptera and Odonata were found exclusively in the diet of rice frogs in Son La Province, whereas Thysanoptera occurred only in the diet of rice frogs in Ninh Binh Province (Pham et al. 2022). The slight differences in the diet between rice frogs in Son La and Ninh Binh may reflect differences in microhabitat use.

As for *O. lingnanica*, the dominant prey items were Coleoptera, Orthoptera, Hymenoptera, Isoptera, and Hemiptera. Of which, beetles (Coleoptera) account for the highest proportion in its food composition. It is also recorded in diet of other populations of frogs (Ngo et al. 2014; Pham and Nguyen 2018; Pham et al. 2019b, 2023, 2024a, 2024b, 2025). Pham et al. (2024c) reported the dietary composition of *Occidozyga martensii*, another representative of the genus from Tay Ninh Province, Vietnam. Both *O. lingnanica* and *O. martensii* inhabit similar environmental conditions, viz. on the ground at the edge of a small slow-flowing stream or puddle in evergreen forest with shrubs (Pham et al. 2024c). The prey categories of *O. martensii* were more diverse than those of *O. lingnanica* (17 vs 13) (Pham et al. 2024c). Interestingly, the prey categories of *O. lingnanica* are similar to those of *O. martensii*, with Coleoptera, Hymenoptera, Orthoptera, and Isoptera representing the most important prey items (Pham et al. 2024c). Nonetheless, Isopoda, Thysanoptera, and Gobiiformes were found exclusively in the diet of *O. lingnanica*, whereas Copepoda, Scolopendromorpha, Blattodea, Dermaptera, Thysanura, Trichoptera, and Anura occurred only in the diet of *O. martensii* (Pham et al. 2024c). The differences in the diet between *O. lingnanica* and *O. martensii* may indicate differences in foraging strategy, microhabitat use, and sites (one in northern – and one in southern Vietnam).

On the other hand, *O. lingnanica* has a more diverse diet and a lower overlap of trophic niche with *M. heymonsi* and *M. mukhlesuri*. Atyidae, Armadillidiidae, Dytiscidae, Staphylinidae, Tenebrionidae, larvae DipteraDrosophilidae, Culicoidea, Cicadellidae, Gerridae, Pentatomidae, Apidae, larvae Lepidoptera, Acrididae, Gryllidae, and Gobiidae were found exclusively in the diet of *O. lingnanica*. However, we also found a slight similarity in the dietary composition of *O. lingnanica* versus *M. heymonsi*, *M. mukhlesuri*. Coleoptera, Hymenoptera, and Isoptera were detected in the diet of three species. In addition, the prey volume of *O. lingnanica* was greater than those the *M. heymonsi* and *M. mukhlesuri* because *O. lingnanica* with larger body sizes, of likely consumes larger prey items than *M. heymonsi* and *M. mukhlesuri* (Forsman 1996; Pham et al. 2023, 2024b).

In Kim Bang SHCA, Ninh Binh Province, Vietnam, *M. mukhlesuri*, *M. heymonsi*, and *O. lingnanica* were found in the same stream habitats or small puddle near the streams, often hid in the leaf litter or small caves during the day and foraged on vegetation or on the ground in the forest floor. The surrounding habitat was the secondary forest, composed of small hardwoods, lianas, and shrubs. However, individuals of *M. heymonsi* and *M. mukhlesuri* were often detected on the ground (18 individuals of *M. heymonsi* /29 individuals of *M. mukhlesuri*), lawn or carpet of fallen leaves (6/8, respectively), in the bank of a stream (0/2, respectively) or puddles (2/3, respectively), with 0.5 to 4.5 meters from the water’s edge, whereas individuals of *O. lingnanica* were found on the on the ground (36 individuals), in leaf litter (9) or muddy areas (3), ~ 0.0–1.0 m from the water’s edge at the stream edge. Such differences in preferred microhabitats and foraging sites they occupy may account for the moderate overlap in the trophic niches between *O. lingnanica* and the two *Microhyla* species. In particular, *O. lingnanica* was usually observed searching for food along the streams, may explain why its diet contains aquatic animals, such as Atyidae and Gobiidae. Meanwhile, *M. heymonsi* and *M. mukhlesuri* consume mainly terrestrial prey like Formicidae, Termitidae, and Phalacridae.

## Additional information

### Artificial Intelligence (AI) use

The authors accept full responsibility for the content of the manuscript, including the disclosure of any use of AI.

No AI tools were used in the preparation of this manuscript.

### Author ORCIDs

V.H.T. Nguyen https://orcid.org/0000-0002-3744-3054

C.T. Pham https://orcid.org/0000-0001-5158-4526

T.Q. Nguyen https://orcid.org/0000-0002-6601-0880

M. Le https://orcid.org/0000-0002-2953-2815

M.D. Le https://orcid.org/0009-0000-5097-8531

A.V. Pham https://orcid.org/0000-0002-6023-3418

### Data availability

All of the data that support the findings of this study are available in the main text.
